# Expression and secretion of fungal endoglucanase II and chimeric cellobiohydrolase I in the oleaginous yeast *Lipomyces starkeyi*

**DOI:** 10.1186/s12934-017-0742-5

**Published:** 2017-07-24

**Authors:** Qi Xu, Eric P. Knoshaug, Wei Wang, Markus Alahuhta, John O. Baker, Shihui Yang, Todd Vander Wall, Stephen R. Decker, Michael E. Himmel, Min Zhang, Hui Wei

**Affiliations:** 1 Biosciences Center, National Renewable Energy Laboratory, Golden, CO 80401 USA; 2 National Bioenergy Center, National Renewable Energy Laboratory, Golden, CO 80401 USA; 3 Hubei Collaborative Innovation Center for Green Transformation of Bio-resources, Hubei Key Laboratory of Industrial Biotechnology, College of Life Sciences, Hubei University, Wuhan, 430062 People’s Republic of China

**Keywords:** *Lipomyces starkeyi*, Oleaginous yeast, Heterologous expression, Cellulase, Cellobiohydrolase I, Endoglucanase II, Advanced biofuels, Consolidated bioprocessing

## Abstract

**Background:**

*Lipomyces starkeyi* is one of the leading lipid-producing microorganisms reported to date; its genetic transformation was only recently reported. Our aim is to engineer *L. starkeyi* to serve in consolidated bioprocessing (CBP) to produce lipid or fatty acid-related biofuels directly from abundant and low-cost lignocellulosic substrates.

**Results:**

To evaluate *L. starkeyi* in this role, we first conducted a genome analysis, which revealed the absence of key endo- and exocellulases in this yeast, prompting us to select and screen four signal peptides for their suitability for the overexpression and secretion of cellulase genes. To compensate for the cellulase deficiency, we chose two prominent cellulases, *Trichoderma reesei* endoglucanase II (EG II) and a chimeric cellobiohydrolase I (TeTrCBH I) formed by fusion of the catalytic domain from *Talaromyces emersonii* CBH I with the linker peptide and cellulose-binding domain from *T. reesei* CBH I. The systematically tested signal peptides included three peptides from native *L. starkeyi* and one from *Yarrowia lipolytica*. We found that all four signal peptides permitted secretion of active EG II. We also determined that three of these signal peptides worked for expression of the chimeric CBH I; suggesting that our design criteria for selecting these signal peptides was effective. Encouragingly, the *Y. lipolytica* signal peptide was able to efficiently guide secretion of the chimeric TeTrCBH I protein from *L. starkeyi*. The purified chimeric TeTrCBH I showed high activity against the cellulose in pretreated corn stover and the purified EG II showed high endocellulase activity measured by the CELLG3 (Megazyme) method.

**Conclusions:**

Our results suggest that *L. starkeyi* is capable of expressing and secreting core fungal cellulases. Moreover, the purified EG II and chimeric TeTrCBH I displayed significant and potentially useful enzymatic activities, demonstrating that engineered *L. starkeyi* has the potential to function as an oleaginous CBP strain for biofuel production. The effectiveness of the tested secretion signals will also benefit future secretion of other heterologous proteins in *L. starkeyi* and, given the effectiveness of the cross-genus secretion signal, possibly other oleaginous yeasts as well.

**Electronic supplementary material:**

The online version of this article (doi:10.1186/s12934-017-0742-5) contains supplementary material, which is available to authorized users.

## Background

The development of biofuels from lignocelluloses is not only important for providing alternative energy to cut the consumption of fossil fuels, but also for sustaining the environment. However, the cost of cellulosic biofuels using current simultaneous saccharification and fermentation (SSF) technology is too high for commercialization, which is a direct consequence of the recalcitrance of biomass to conversion to fermentable sugars [1]. Consolidated bioprocessing (CBP) represents a more recent process design wherein lignocellulosic feedstocks are deconstructed and converted to biofuels in a single tank using a single microorganism [2–4]. Several bacterial, yeast, and filamentous fungal organisms have been explored as CBP strains to produce biofuels [5–11], with *Lipomyces starkeyi* emerging as a new candidate.

*Lipomyces starkeyi* is a notable oleaginous yeast because its lipid content can reach more that 70% of the cell dry weight. Additionally, it can utilize glucose, xylose, mannose, and cellobiose to produce lipids [12–15], and its lipid fractions are dominated by oleic and linoleic acids (ideal precursors for jet fuels [15]). Its genetic transformation systems have been established [16, 17]. However, *L. starkeyi* can produce high-yield lipids only from soluble sugars and cannot digest and utilize lignocelluloses directly. To reduce the cost of lipids or lipid-related hydrocarbons produced by *L. starkeyi*, we sought to express key fungal cellulases in *L. starkeyi*.

To date, a considerable number of bacterial and fungal glycoside hydrolase genes have been expressed in yeast to constitute either free cellulase systems or designed “mini-cellulosomes” [3, 18–22]. Despite this progress, none of these engineered yeast strains were able to express cellulases that achieved the titers and/or activities comparable to the source species, such as *Trichoderma reesei* [23]. This poor performance may be due to incorrect folding [24] or hyperglycosylation of the recombinant proteins [25–32]. Another possible reason for the poor cellulase performance observed so far is that suitable signal peptides are also important for cellulase expression in yeast, as they guide proteins to and through the endoplasmic reticulum and the secretory pathway [33–35]. Indeed, some specific signal peptides have been shown to enhance the secretion efficiency of targeted proteins, including heterologous cellulases [36–39], whereas others can affect the functionality of secreted cellulases [40]. More recently, some cellulase and hemicellulase genes introduced to oleaginous yeasts resulted in relatively high activity and yield of secreted heterologous proteins [41–45].

The objective of our work is to explore the suitability of *L. starkeyi* as a CBP platform microorganism. We first performed a genome wide search of *L. starkeyi* for endogenous cellulases, including β-d-glucosidases. Secondly, we combined a set of four signal peptides and two key fungal cellulase genes and screened for expression and activity. The four signal peptides included spPRO (*Yarrowia lipolytica*), and spAMY, spDEX1, spDEX2 (*L. starkeyi*) as described later. The cellulases included a chimeric CBH I formed by fusing the catalytic domain from *Talaromyces emersonii* CBH I with the linker peptide and cellulose-binding domain of *T. reesei* CBH I (TeTrCBH I) and *T. reesei* EGII [41]. To the best of our knowledge, this is the first study to show that fungal cellulases, chimeric CBH I and EG II, can be efficiently expressed in *L. starkeyi*, rendering it a potential CBP strain for biofuels production.

## Results

### Genome-wide search for endogenous cellulases in *L. starkeyi*

The genome of *L. starkeyi* NRRL Y-11557 v1.0 was recently released by the JGI (http://genome.jgi.doe.gov/Lipst1_1/Lipst1_1.home.html). In total, 8192 sequences for putative proteins were annotated (see Additional file 1: Table S1, Sheet 1). Based on this *L. starkeyi* genome sequence analysis, the putative genes related to lignocellulose degradation and utilization were identified (Additional file 1: Table S1, Sheet 2, Column I). Genes related to enzymes that digest starch, such as amylase and dextranases, have been characterized [46–48]. Although some putative endoglucanase-encoding genes have been identified in the genome sequence, genes for the key cellulases including endoglucanases and cellobiohydrolases (CBH) needed for depolymerizing cellulose to fermentable sugars, such as glycoside hydrolase families (GH) 5, 6, 7, and 9, have not been identified (Additional file 1: Table S1, Sheet 2, Column I). Encouragingly, *L. starkeyi* has been reported to co-ferment cellobiose and xylose [12], suggesting the presence of β-glucosidases (either extracellular β-glucosidases, and/or intracellular β-glucosidases working in tandem with a cellobiose-transporter or transporters). Consistent with that report, our genomic analysis revealed the existence of two putative β-glucosidase genes in the genome sequence (jgi|Lipst1_1|147|gm1.147_g and jgi|Lipst1_1|5081|gm1.5081_g), both of them belong to GH3 (Additional file 1: Table S1, Sheet 2).

In our first attempt to engineer *L. starkeyi* for CBP, the above analysis prompted us to focus on transforming *L. starkeyi* cells with constructs containing two key cellulase genes, starting with selecting the signal peptides needed to express and secrete the heterologous cellulases in *L. starkeyi*.

### Signal peptide selection

Signal peptides play a key role in the secretion of targeted proteins in microorganisms. A thorough description of the roles signal peptides play in the protein secretion of yeast can be found in a review [49]. After the proteins are synthesized and released from ribosomes, the hydrophobic core of the secretory signal peptide of proteins either interact with the signal recognition particle (SRP) in cytosol, or bind with cytosolic chaperones and their cofactors, and then the proteins are transferred to the translocon channel in the ER membrane through the co- or post-translational translocation routes in the early secretory pathway. The pre- and pro-cleavage sites in the signal peptides are cleaved in the ER and Golgi apparatus, respectively, during trafficking. Eventually, the signal peptide-cleaved proteins are transported from Golgi to the cell surface, and the mature proteins are released extracellularly. The choice of different signal peptides has been shown to significantly affect the yield of secreted proteins, including cellulases [40]. Thus, screening signal peptides is an important strategy for optimizing the secretion of heterologous proteins in bacteria and yeasts [50–52]. We implemented a signal peptide screening approach for expressing cellulase genes in *L. starkeyi* based on the following criteria: signal peptides used for prominent proteins in the secretome of *L. starkeyi* or signal peptides used for overexpressing cellulose genes in other yeasts such as *Y. lipolytica*.

The four signal peptides chosen for this study are summarized in Table 2. These signal peptides can be divided into two groups: one group is from a different yeast, *Y. lipolytica* (the alkaline extracellular protease, spPRO), and the other group is from native *L. starkeyi* NRRL Y-11557, including signal peptides for the amylase, dextranase 1, and dextranase 2 genes (spAMY, spDEX1 and spDEX2, respectively). The length of these signal peptides ranged from 16 to 32 residues.

### Expression of chimeric CBH I and EG II in *L. starkeyi* using four selected signal peptides

**Fig. 1:**
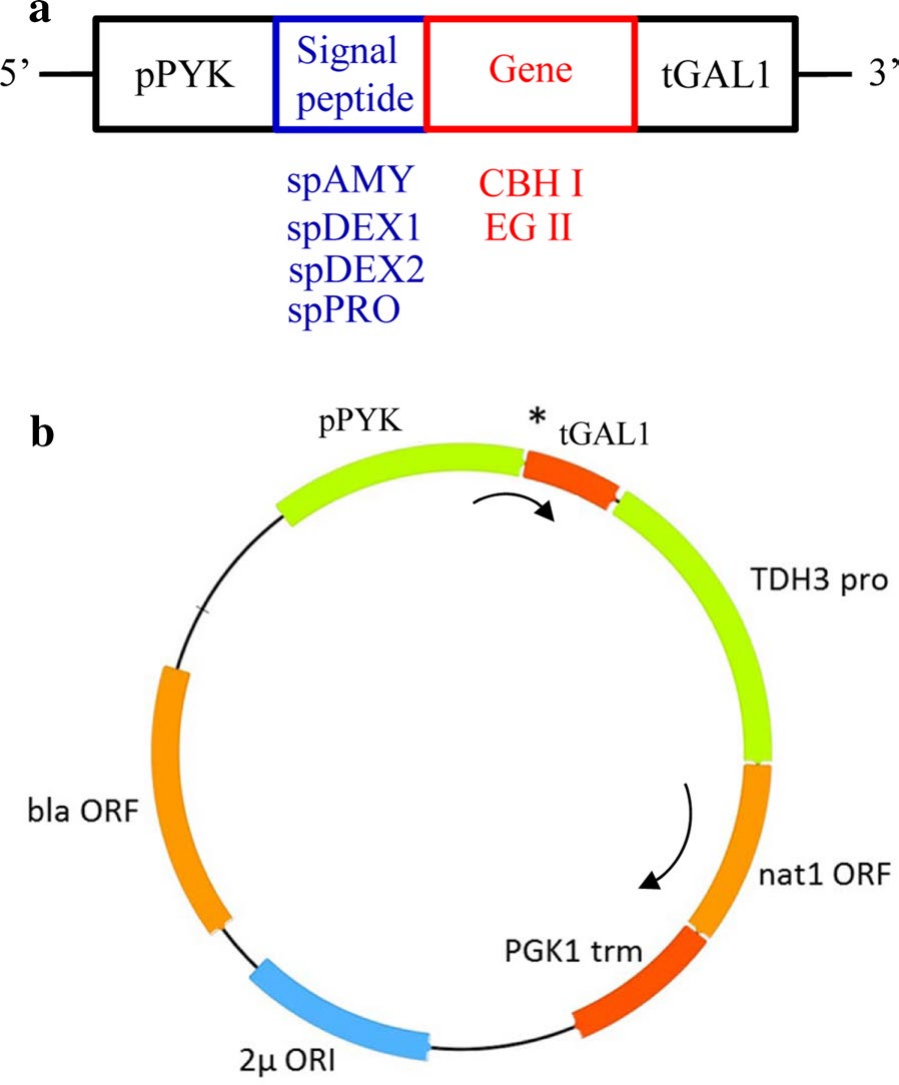
Diagram of gene cassettes and vector used for cellulase expression in *L. starkeyi.* **a** Cellulase gene expression cassette; **b** the base vector, in which the *star* denotes where genes of interest are cloned into. Bla, beta-lactamase gene for ampicillin resistance; PGK1 trm, 3-phosphoglycerate kinase gene’s terminator; pPYK, promoter of *L. starkeyi pyk* (pyruvate kinase gene); spAMY, signal peptide of *L. starkeyi* amylase; spDEX1, signal peptide of *L. starkeyi* dextranase 1; spDEX2, signal peptide of *L. starkeyi* dextranase 2; spPRO, signal peptide of *Y. lipolytica* protease; TDH3 pro, glyceraldehyde-3-phosphate dehydrogenase gene’s promoter; tGAL1, terminator of *L. starkeyi gal1* (galactokinase 1 gene); 2µ, *S. cerevisiae* 2-micron origin of replication. Gene, cellulase gene without native signal peptide. Size of various signal peptides is described in Table 2, while the size of chimeric CBH I and EG II is 500 and 397 in amino acid residues, respectively; the size of fusion genes that encode signal peptide-cellulase can be deduced accordingly

The selected signal peptides were fused to the catalytic domains of the cellulase genes, chimeric TeTr*cbh1* and *eg2,* respectively (Fig. 1a). These constructs were cloned into expression cassettes for *L. starkeyi* containing native pyruvate kinase (*pyk*) promoter and the galactokinase (*gal1*) terminator, as illustrated in Fig. 1b; the selection marker used was the nourseothricin acetyltransferase (nat1) from *Streptomyces noursei* giving resistance to nourseothricin (clonNAT). The resultant plasmids were linearized and transformed into *L. starkeyi* NRRL Y-11557 to produce recombinant cellulase proteins.

Random and targeted integration genetic transformation systems for *L. starkeyi* have recently been reported [16, 17]. In this study, *L. starkeyi* NRRL Y-11557 transformation efficiency ranged from 3 to 82 colonies per µg of linearized plasmid DNA (Table 3), demonstrating the successful utilization of the random integration transformation system. However, the achieved transformation efficiency in our study was much lower than that of 8000 colonies per µg of linearized plasmid DNA reported by Calvey et al. [16]. It is unclear why our transformation efficiencies were much lower compared to the reference, and future studies are needed to optimize the variables in order to achieve higher transformation efficiencies.

Literature has shown that the levels of heterologous protein expression had been reported to be significantly affected by the different locations for the integration of transgenes into the genome of host organisms [53–55]. Specifically, by using a random integration approach, an up to 14-fold difference was observed between the lowest and highest β-galactosidase expression in 18 random lacZ insertion transformants of yeast [55]. Thus, in general multiple transformants needed to be screened for either the production level of heterologous proteins or their desired phenotypes in the genetic transformation studies of eukaryotic organisms [56–58].

**Fig. 2:**
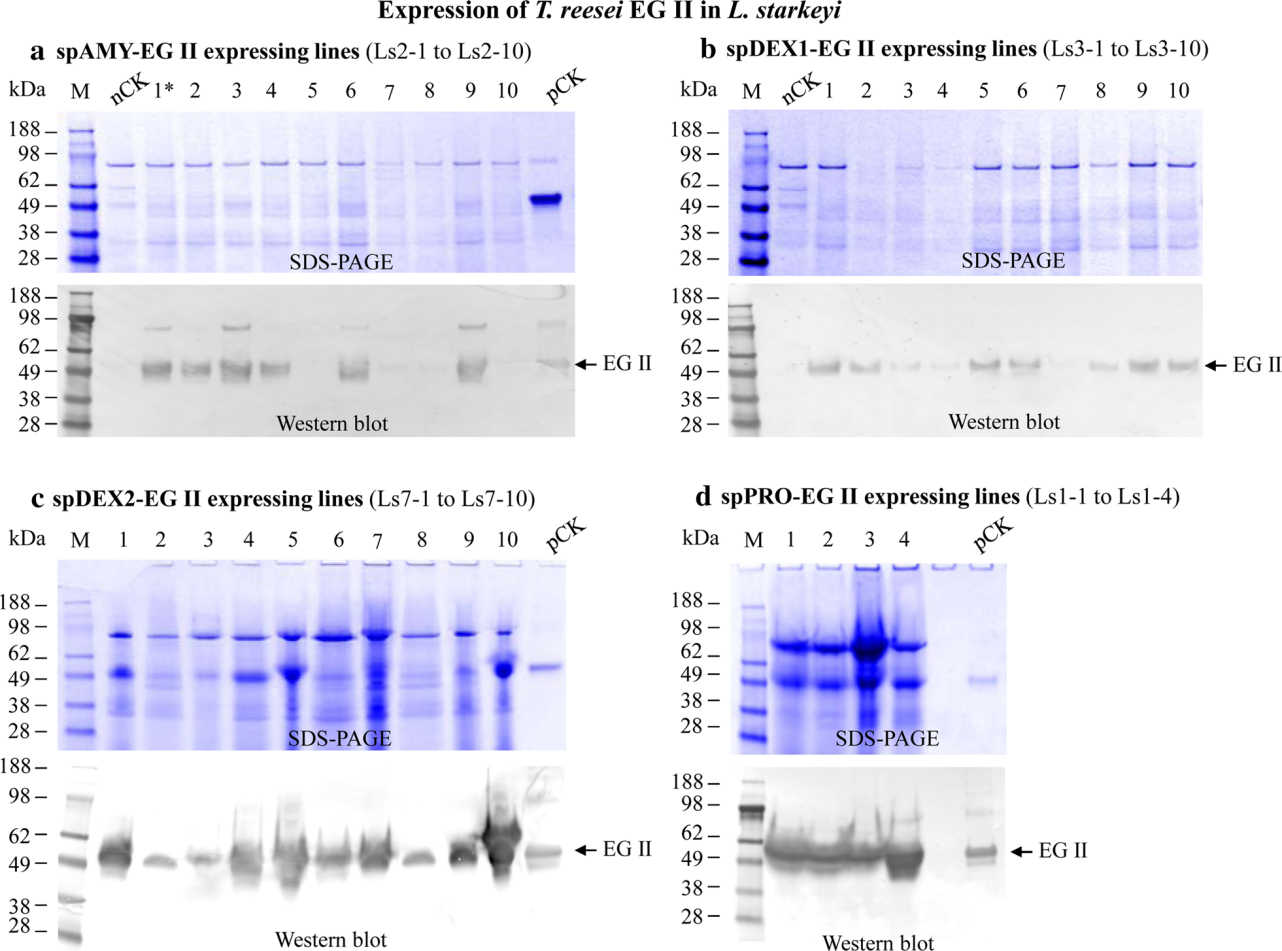
SDS-PAGE and western blot analysis of the overexpression of *T. reesei eg2* gene in *L. starkeyi* guided by signal peptides derived from *L. starkeyi* amylase gene, dextranase 1 or 2 genes, or *Y. lipolytica* protease gene. **a** Signal peptide of *L. starkeyi* amylase (spAMY); *lanes 1–10*, exoproteome produced by transformants integrated with plasmid pLS2-2; *star* indicates the transformant with the highest level of EG II secretion in this subset of strains. **b** Signal peptide of *L. starkeyi* dextranase 1 (spDEX1); *lanes 1–10*, exoproteome produced by transformants integrated with plasmid pLS3-1. **c** Signal peptide of *L. starkeyi* dextranase 2 (spDEX2); *lanes 1–10*, exoproteome produced by transformants integrated with plasmid pLS7-1. **d** Signal peptide of *Y. lipolytica* protease (spPRO); *lanes 1–4*, exoproteome produced by transformants integrated with plasmid pLS1-3. For the samples in each of four *panels* (**a**–**d**), nCK, negative control, exproteome produced by transformant integrated with empty vector; pCK, positive control, purified *T. reesei* EGII protein. Culture supernatant was concentrated 2× for **a** and **b**, or 35× for **c** and **d**

**Fig. 3:**
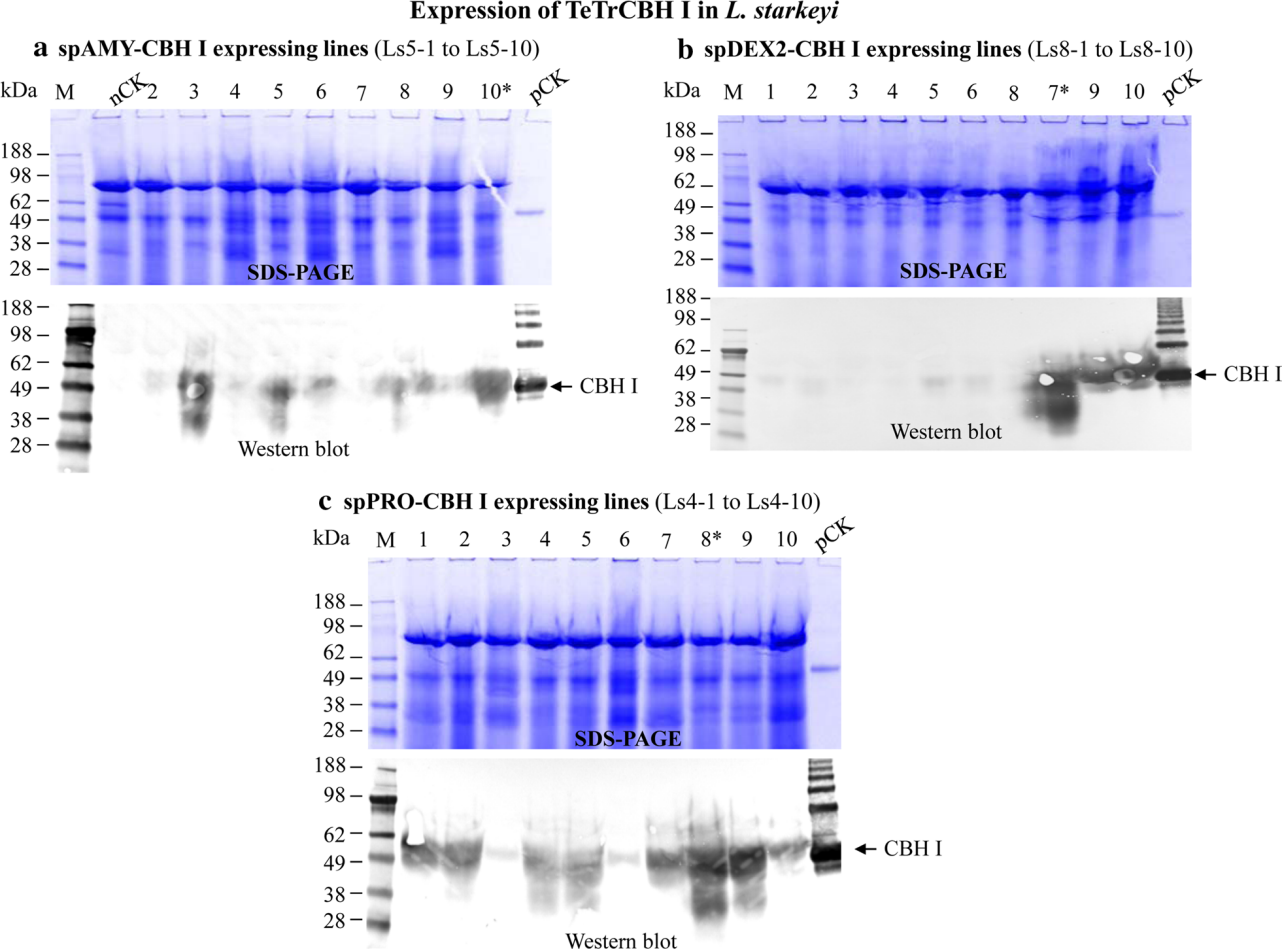
SDS-PAGE and western blot analysis of the overexpression of chimeric *TeTr*CBH I in *L. starkeyi* guided by signal peptides of *L. starkeyi* amylase or dextranase 2 gene, or *Y. lipolytica* protease gene. **a** Signal peptide of *L. starkeyi* amylase (spAMY); *lanes 2–10*, exoproteome produced by transformants integrated with plasmid pLS5-6. **b** Signal peptide of *L. starkeyi* dextranase 2 (spDEX2); *lanes 1–10*, exoproteome produced by transformants integrated with plasmid pLS8-2. **c** Signal peptide of *Y. lipolytica* protease (spPRO); *lanes 1–10*, exoproteome produced by transformants integrated with plasmid pLS4-1. For the samples in each of four *panels* (**a**–**c**), nCK, negative control, exoproteome produced by transformant integrated with empty vector; pCK, positive control, purified *T. reesei* CBH I protein. Culture supernatant was concentrated by 35×. *Star* indicates the transformants with the highest level of CBH I secretion in each subset of strains

Transformants with integrated cellulase genes were cultured and their supernatants were concentrated 35 or two times by using Vivaspin 20 10-kDa concentrators, and washed by refilling the concentrator with PBS buffer and re-concentrating to appropriate volume. The rationale for concentrating supernatants by 35 times was to follow our previous procedure used for characterizing the *Y. lipolytica* transformants that expressing cellulases [41]. Such procedure worked well for examining the *L. starkeyi* transformants that expressed either spDEX2-EG II and spPRO-EG II) (Fig. 2c, d), or chimeric CBH I (Fig. 3). However, we noticed that the strong western blot bands in Fig. 2c, d seem to suggest that the amount of EG II proteins loaded into the SDS-PAGE wells might be too high when the 35× concentration preparations were used. Such observation prompted us to test the use of 2× concentration preparations for the western blot analysis of the remaining *L. starkeyi* transformants that expressed spAMY-EG II and spDEX1-EG II. Remarkably, the western blot using 2× concentration preparations of proteins showed that the signals for detected EG II bands were strong enough for an efficient screening of the transformants (Fig. 2a, b), which should benefit future studies.

The concentrated supernatants described above were analyzed by SDS-PAGE, followed by visualization using western blot with their respective cellulase antibodies. Based on positive western blot results, we demonstrated that all four signal peptides described above performed well for secretion of the recombinant EG II protein (Fig. 2), and three of the tested signal peptides also worked well for secretion of TeTrCBH I (Fig. 2; Table 2). Note that the fourth signal peptide was not further tested as successful solutions were found with the other three.

It was observed that the cellulase protein yields in the exoproteome produced by various transformants with the same signal peptide varied by western blot intensity (see the individual panels in Figs. 2, 3). For example, using the signal peptide of the *L. starkeyi* amylase gene (spAMY) to guide the chimeric CBH I secretion, two of the nine transformants showed very strong intensity by western blot, whereas the other seven transformants exhibited low or no intensity (Fig. 2a). This result was likely due to the effects of random insertion and/or different copy numbers of target gene integration into the host genome. The method of integration for transformation of *L. starkeyi* is presumably non-homologous end joining as we did not add any homologous sequences to our constructs.

### Effect of signal peptides on cellulase secretion efficiency

**Fig. 4:**
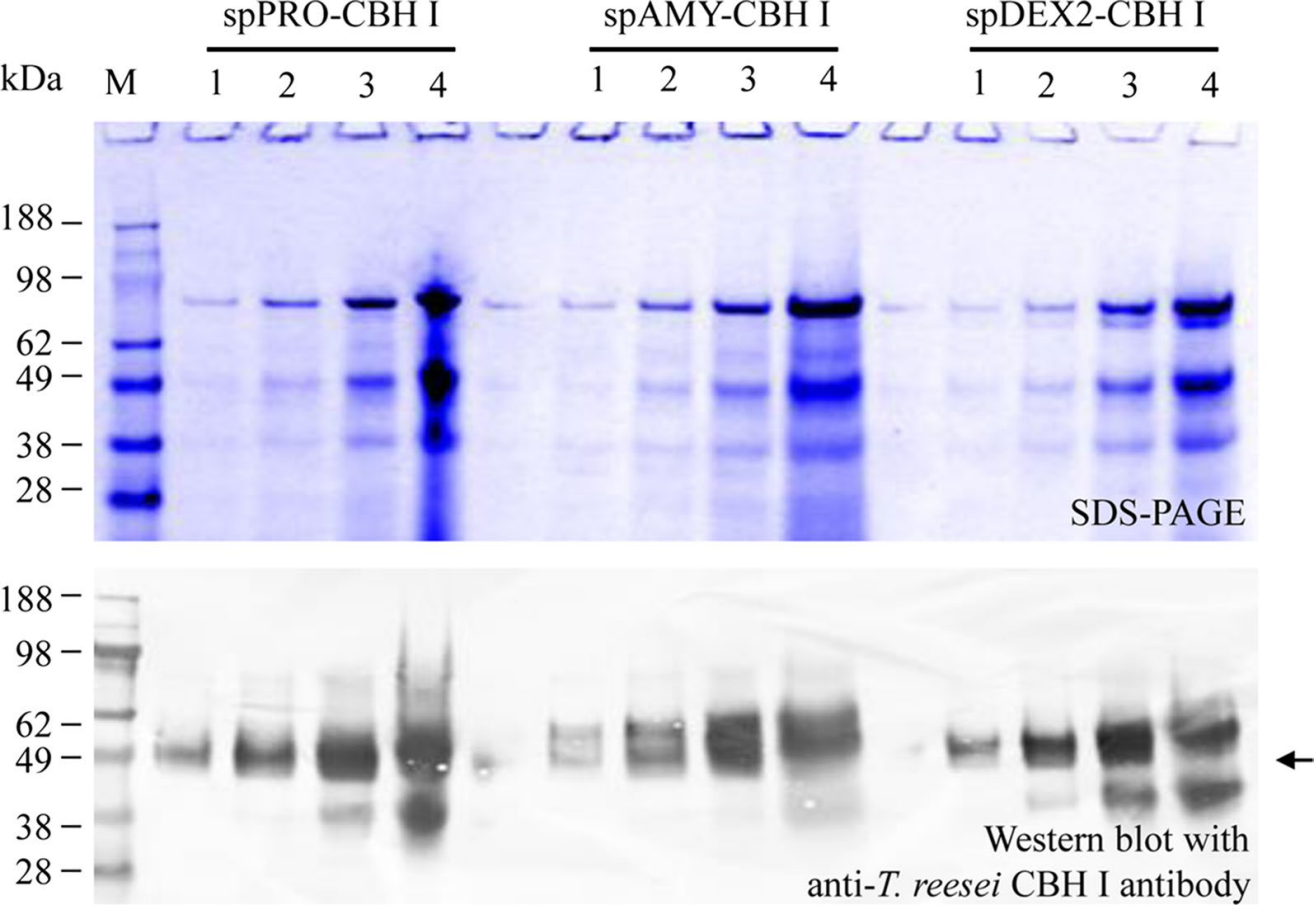
Comparison of the secretion efficiency of the chimeric *TeTr*CBH I protein expressed in *L. starkeyi* guided by three different signal peptides. Under the category of each signal peptide, *lanes 1–4* represent the supernatants that were diluted by 27, 9, 3 and 1×, respectively. Culture supernatant was concentrated 35× first, and then diluted in series as indicated above. All diluted proteins were analyzed by SDS-PAGE and detected by western blot. spPRO, signal peptide of *Y. lipolytica* protease; spAMY, signal peptide of *L. starkeyi* amylase; spDEX2, signal peptide of *L. starkeyi* dextranase 2

To compare the secretion efficiency for the selected signal peptides, one representative transformant was selected from those shown in each of the three panels in Fig. 3 to represent transformations with a specific signal peptide, i.e., spPRO-CBH I, spAMY-CBH I, and spDEX2-CBH I. These representative transformants were selected and cultured, and their culture supernatants were concentrated and washed, and diluted in series. These proteins were analyzed by SDS-PAGE and analyzed by western blot in parallel (Fig. 4). The intensity of the western blot bands for these three transformants was similar, indicating that the secretion efficiency of these three signal peptides from the *Y. lipolytica* protease (spPRO), *L. starkeyi* amylase (spAMY), and dextranase II (spDEX2) genes were similar. Indeed, all of these constructs led to high chimeric CBH I production. Surprisingly, the secretion efficiency of the *Y. lipolytica* signal peptide (spPRO) in *L. starkeyi* is similar to that of the native *L. starkeyi* signal peptides (spAMY and spDEX2); which suggests that the secretory pathway has a high level of cross-genus conservation and that additional signal peptides from other yeast or filamentous fungi should be tested for potentially enhanced secretion of recombinant proteins produced in *L. starkeyi*.

### Distribution of chimeric CBH I in *L. starkeyi* transformant cells

**Fig. 5:**
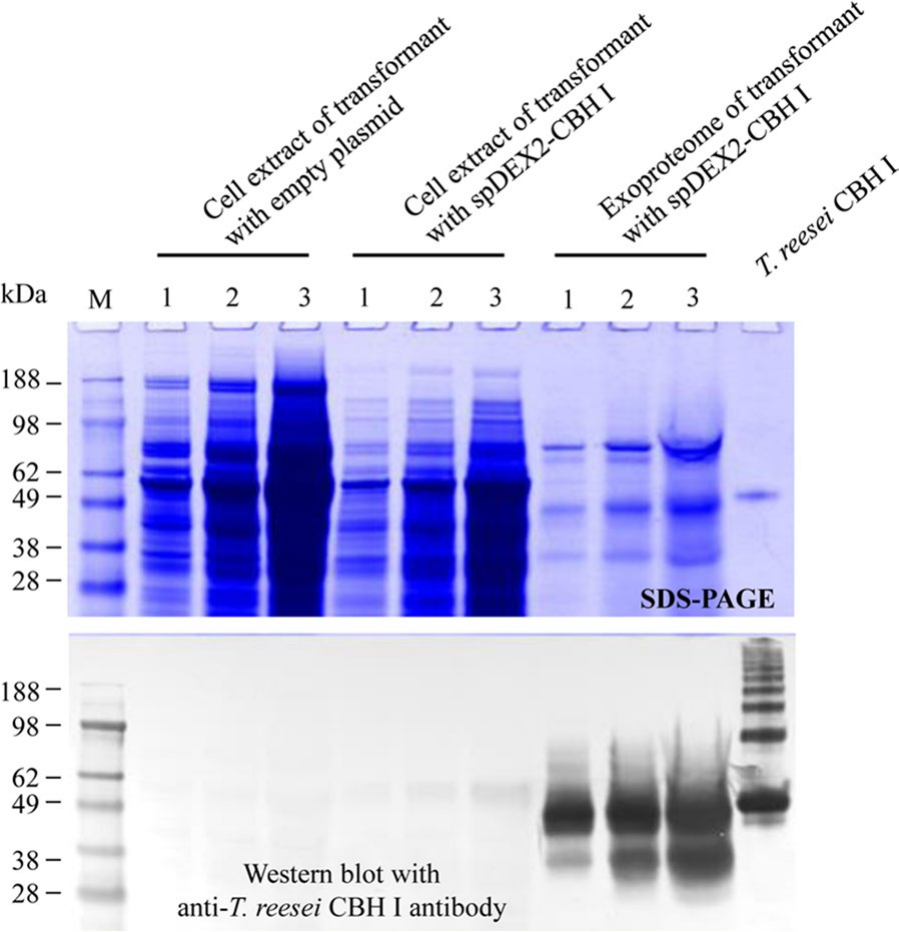
SDS-Page and western blot analysis of intracellular versus extracellular secretion of the chimeric TeTrCBH I protein in *L. starkeyi.* The strain of LS8-7 with signal peptide from *L. starkeyi* dextranase 2 gene was cultured for this analysis. *Lanes 1–3* indicate cell extracts or exoproteomes that were diluted to 9, 3 and 1×, respectively. For cell extracts, the protein was extracted from cell pellets of culture and diluted in series as indicated above. For exoproteomes, culture supernatant was concentrated 35× and diluted in series

To investigate the secretion efficiency of chimeric CBH I guided by the signal peptide from *L. starkeyi* dextranase II (spDEX2), the proteins from cell pellets and supernatants derived from selected transformants of *L. starkeyi* were characterized by SDS-PAGE and western blots (Fig. 5). Bands positive for CBH I revealed by western blot of intracellular extracted proteins from the CBH I transformant were very faint and similar to those of the empty vector transformant; whereas the bands positive for CBH I from the culture supernatant were very strong. These results demonstrated that almost all synthesized chimeric CBH I protein guided by the signal peptide derived from *L. starkeyi* dextranase II gene was efficiently secreted into medium.

### Copy numbers for top chimeric CBH I- and EGII-producing transformants

The western blot results (Figs. 2, 3) allowed us to pick the transformants with the highest relative secretion level for TeTrCBH I or EG II, from each set of transformants with different secretion signal peptides. As indicated by the star marks in Figs. 2a and 3a–c, the top spAMY-EG II expressing transformant was LS2-1, while the top spAMY-, spDEX2- and spPRO-CBH I expressing transformants were Ls5-10, Ls8-7 and Ls4-8, respectively.

From each of the above transformants, the genomic DNA was isolated from cell pellets, serial-diluted, and used to generate relative standard curves for the target genes (either cbh I or eg II) as well as the single-copy reference gene eif5, using the primers listed in Additional file 2: Table S2. The obtained slope, intercept, R^2^, and the calculated amplification efficiency (E) are listed in Additional file 2: Table S3. The E values for EIF5 and CBH I primers were in the range of 0.89–0.99 and 0.92–0.99, respectively, while the E value for EG II primers was 0.91. The copy number (X_0_/R_0_) for each transformant was calculated by following the methodology of Weng et al. [59], as outlined in “Methods” section. The results showed that for CBH I transformants, lines Ls4-8 (X_0_/R_0_ = 0.90 ± 0.12) and Ls8-7 (X_0_/R_0_ = 0.92 ± 0.03) harbored single copies of the TeTrCBH II transgene, while line Ls5-10 (X_0_/R_0_ = 1.72 ± 0.20) harbored two copies. For EG II transformant Ls2-1 (X_0_/R_0_ = 0.93 ± 0.05), it harbored single copy of the EG II transgene.

### Enzyme activity analysis of the top TrTeCBH I-producing transformants

The top spAMY-, spDEX2- and spPRO-CBH I expressing transformants, namely Ls5-10, Ls8-7 and Ls4-8, were used to conduct enzyme activity analysis of crude enzymes (secretome), which were prepared as described in “Methods” section. The crude enzymes (secretome) instead of purified TeTrCBH I were used based on the rationale that the secretome is more closely related to the real-world scenario of a CBP strain’s utilization of cellulosic substrates, by which it is a mixture of secreted proteins (containing the expressed TeTrCBH I) acting on the cellulosic substrate. The “empty-vector (EV) -transformant” strain’s secretome was used as the control.

The results, as presented in Additional file 2: Figure S1, clearly shows that all three of the secretomes produced by strains transformed with TeTrCBH I have significantly higher cellulase activity than the secretome from the empty-vector transformant. Most, if not all, of the activity shown for the empty-vector transformant can be attributed to the standard loadings of β-glucosidase and E1-CD endoglucanase “helper” enzymes that were added to the assays of all transformants to provide an estimate of the performance of ach CBH I as part of a full, synergistic cellulase system. It is further evident that the CBH I transformants fall into two groups as far as activity is concerned, with LS5-10 transformant showing roughly twice the activity of the other two. This difference is not at all surprising given that, as detailed above, LS5-10 has been shown to carry two copies of the TrTeCBH I gene, whereas LS8-7 and LS4-8 each carry one copy of the gene.

### Endoglucanase activity of the purified recombinant EG II secreted in *L. starkeyi*

The recombinant EG II secreted from *L. starkeyi* was purified and activity was measured by the CELLG3 endoglucanase assay kit (Megazyme, Bray, Iceland). The CELLG3 activity of purified EG II was found to be 3.97 ± 0.04 CELLG3 units per mg of the purified protein; one CELLG3 unit is defined as one micromole of 2-chloro-4-nitrophenol released from CELLG3 in one min under the defined assay conditions (see CELLG3 assay kit by Megazyme).

In comparison, the activity of *Acidothermus cellulolyticus* endoglucanase E1 (Y245G), a well-known enzyme used in the CBH I activity assay [41], was also tested and its CELLG3 activity was 2.49 ± 0.10 units per mg of the purified protein. The EG II produced by *L. starkeyi* thus demonstrated an endoglucanase activity (as measured by the CELLG3 kit) 59% higher than that of *A. cellulolyticus* E1, suggesting the proper folding and secretion signal cleavage of the expressed EGII in *L. starkeyi*.

### Cellobiohydrolase (CBH) activity of the purified chimeric TeTrCBH I secreted in *L. starkeyi*

**Fig. 6:**
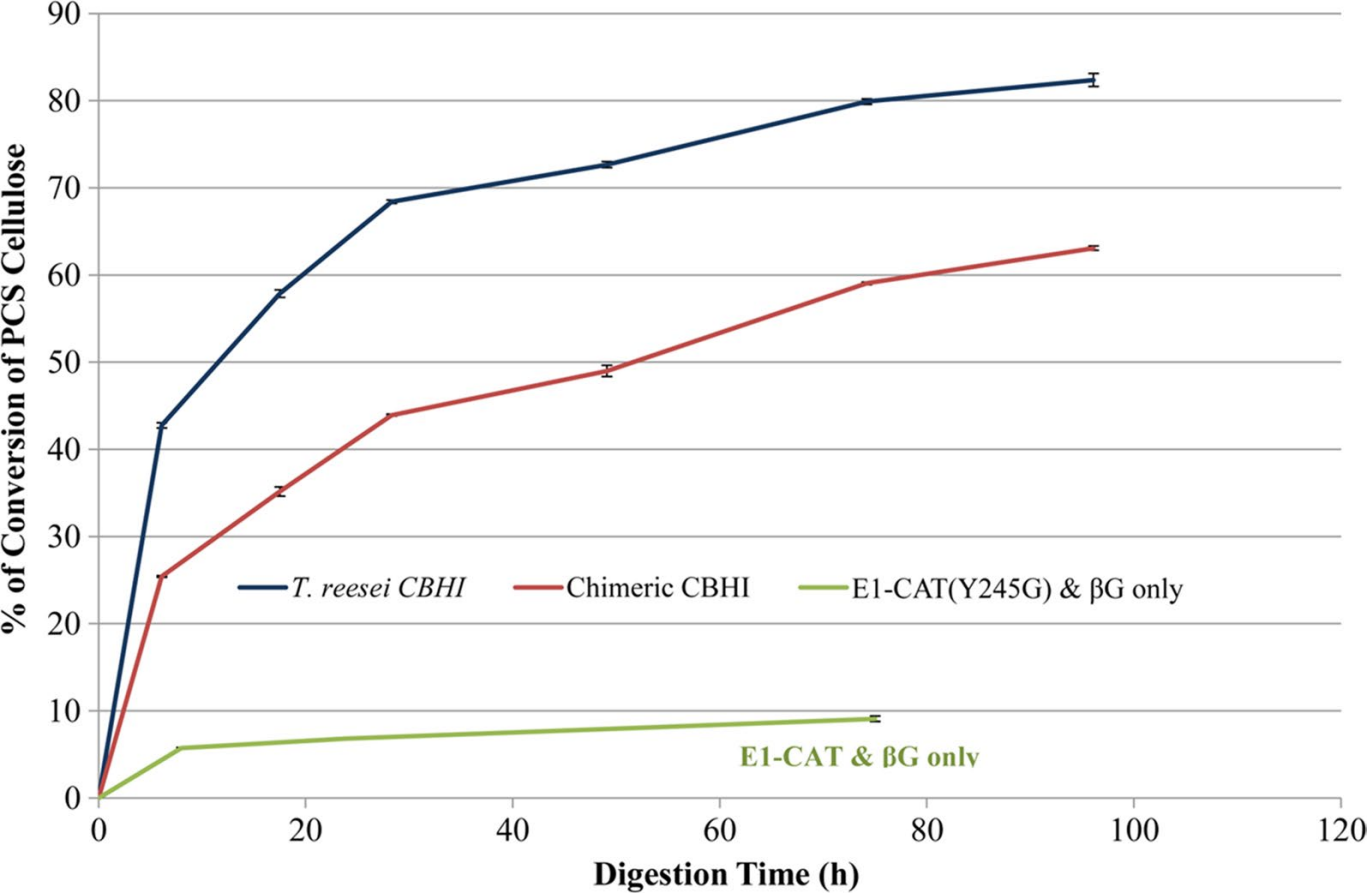
Cellulolytic activities of purified *T. reesei* CBH I secreted by *T. reesei* and chimeric *TeTr*CBH I secreted by *L. starkeyi*. The enzyme cocktails consisted of CBH I (either *T. reesei* CBH I or chimeric TeTrCBH I; 28 mg/g substrate), *A. cellulolyticus* E1-CAT (Y245G) (1.89 mg/g substrate) and *A. niger* β-d-glucosidase (0.50 mg/g substrate). The substrate used for these assays was pretreated corn stover loaded at 5 mg/mL in 20 mM acetate buffer pH 5.0 and the assay temperature was 40 °C. *T. reesei* CBH I was purified from the native host; chimeric TeTrCBH I is the *Talaromyces emersonii* CBH I catalytic domain fused to the linker and CBM from *T. reesei* CBH I and was expressed in *L. starkeyi* guided by the signal peptide of *L. starkeyi* dextranase II. βG is the β-d-glucosidase purified from *A. niger*; and E1-CAT (Y245G) is the engineered *A. cellulolyticus* endocellulase E1 catalytic domain

The chimeric TeTrCBH I secreted from *L. starkeyi* was purified and its activity in saccharification of a model industrial lignocellulosic substrate was determined, relative to that of TrCBH I expressed in *T. reesei* (Fig. 6). The conversion of pretreated corn stover cellulose by this purified TeTrCBH I reached 63.1% after incubation of 96 h, while conversion by the purified native *T. reesei* CBH I (secreted by *T. reesei*; used as a control here) for the same length of time of digestion reached 82.4% (Fig. 6). Such results indicate that the TeTrCBH I secreted by *L. starkeyi* was able to substantially convert the pretreated corn stover cellulose to cellobiose and glucose, and could be further improved as its conversion of substrate at 96 h was 76.6% of that found for purified, native *T. reesei* CBH I.

### Deglycosylation analysis of chimeric cellobiohydrolase I

**Fig. 7:**
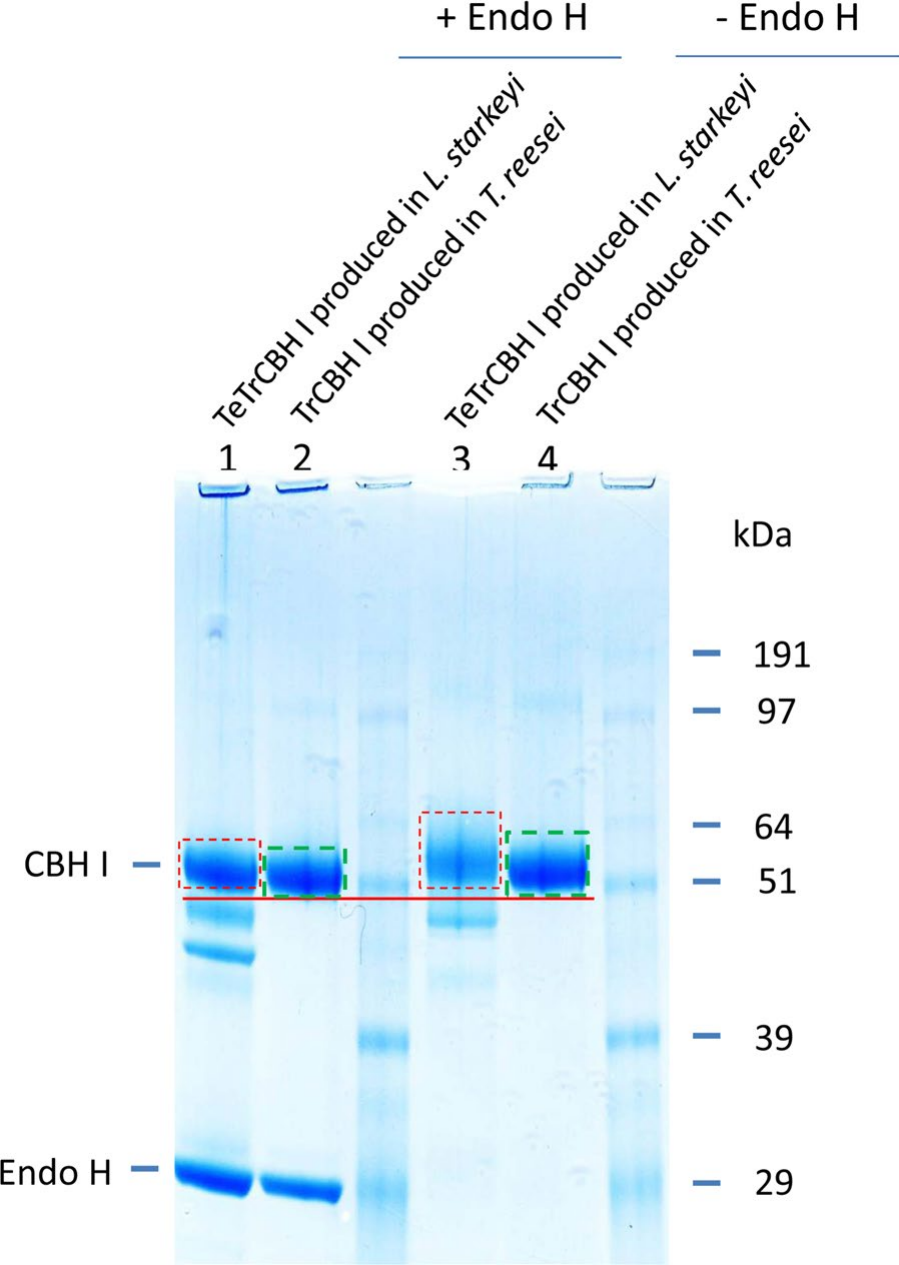
SDS-PAGE of deglycosylated chimeric TeTrCBH I protein expressed in *L. starkeyi* and native TrCBH I produced in *T. reesei* Rut-C30 as control. Protein samples were deglycosylated with Endo H or non-treated (-Endo H). The protein loading was 20 μL (containing 10 μg protein) per well

The purified TeTrCBH I protein produced in *L. starkeyi* was subjected to deglycosylation analysis to determine the extent of its N-linked glycosylation. The protein was digested with endoglycosidase H (Endo H), which cleaved the N-linked oligosaccharide chain in the glycoprotein. The MW difference between protein samples with and without Endo H treatment reflects the extent of protein glycosylation mediated by the expression host. The results demonstrate that for the TeTrCBH I expressed in *L. starkeyi*, its MW on SDS-PAGE was approximately 63 kDa for the glycosylated form (without Endo H treatment; Fig. 7, lane 3) whereas 53 kDa for the deglycosylated form (with Endo H treatment; Fig. 7, lane 1), suggesting that about 10 kDa of N-linked glycan was removed. Similarly, for native CBHI produced in *T. reesei*, its MW on SDS-PAGE was about 61 kDa for the glycosylated form and 52 kDa for the deglycosylated form, suggesting that 9 kDa of N-linked glycan was removed (Fig. 7, lane 4 versus lane 2), which was consistent with our previous analysis for the deglycosylated native TrCBH I [41]. This observation suggested that in the case of TeTrCBH I expressed in yeast *L. starkeyi*, the overall magnitude of glycosylation was higher than that for native CBH I produced in *T. reesei*, which is reflected by the apparently more smeared band of TeTrCBH I without the Endo H treatment (Fig. 7, lane 3, band indicated by the red box), compared to the relative tight band of native TrCBH I (Fig. 7, lane 4, band indicated by the green box). This could partially explain why the percentage of conversion of PCS cellulose by TeTrCBH I was lower than that by native TrCBH I (Fig. 6).

## Discussion

### Secretion of recombinant cellulases guided by signal peptides

Chimeric CBH I and EG II can be expressed and secreted using various signal peptides, both from native *L. starkeyi* and *Y. lipolytica*. Based on our best knowledge, this is the first report showing the successful expression and secretion of heterologous fungal cellulase genes in *L. starkeyi*. It is also encouraging that the *Y. lipolytica* signal peptide can successfully target chimeric CBH I and EG II for secretion in *L. starkeyi*, implying that additional signal peptides may be found from other yeast or filamentous fungal species that may further enhance secretion of heterologous proteins in *L. starkeyi.*

### Towards consolidated bioprocessing

Our eventual goal is to develop *L. starkeyi* as a CBP strain in which biomass saccharification and lipid or hydrocarbons production are combined into a single step. In this approach, critical heterologous cellulase genes are expressed in *L. starkeyi*, and effectively secreted in highly active forms where they convert complex lignocellulosic substrates to soluble sugars for lipid production. More studies related to enhancing *L. starkeyi* cellulolytic capability are needed, including:

#### (1) Stronger promoters

In this study, a single promoter of native *L. starkeyi* was employed for all cellulase overexpression. Current work demonstrated that the chimeric CBH I protein was secreted almost completely into the medium, suggesting that the secretion of this protein is probably not the bottleneck for cellulase protein production. However, efficient protein synthesis may be a critical limitation for CBH I production in *L. starkeyi*. Thus, using a stronger promoter is an important consideration for achieving higher titers of CBH I protein from *L. starkeyi*. To date, two native *L. starkeyi* promoters for glyceraldehyde-3-phosphate dehydrogenase (tdh3) and pyruvate kinase (pyk) gene have been reported for gene expression in *L. starkeyi* [16, 17].

#### (2) Glycosylation

It has been reported that recombinant cellobiohydrolase enzymes in yeast exhibit variable levels of glycosylation. In some cases, hyper-glycosylation resulted in low activity of heterologous cellulases from yeast [24, 25, 30, 60, 61]. In this study, we observed the typical ‘smear’ and shifted bands for the expressed chimeric CBH I protein in the western blots, which implied that the recombinant protein was glycosylated to various extents. However, the impact of the glycosylation on the activities of the recombinant cellulase proteins produced by *L. starkeyi* has not yet been investigated. Future studies are needed to determine whether hyper- or non-native-glycosylation of recombinant cellulase proteins produced in *L. starkeyi* (or yeast in general) affects performance.

## Conclusions

The goal of this study is to engineer *L. starkeyi*, a leading lipid-producing yeast, to serve as a CBP strain for converting lignocellulosic substrates to lipid or fatty acid-related biofuels. Despite the general challenge of expressing fully active fungal CBH I in yeast; as well as the paucity of experimentally tested signal peptides prior-to-this study, we successfully demonstrated the efficient expression, secretion, and function of heterologous *T. reesei* EG II and chimeric TeTrCBH I in *L. starkeyi*. Overall, our results prove the suitability of *L. starkeyi* for expression of core fungal cellulases and lay a foundation for further developing *L. starkeyi* into a cellulolytic, oleaginous CBP organism. Furthermore, the sequences of the tested secretion signals (especially the cross-genus effective one) should be useful in future studies for the expression and secretion of other heterologous proteins in *L. starkeyi* and perhaps other yeast species as well.

## Methods

### Strains and medium

Wild-type (WT) *L. starkeyi* NRRL Y-11557 used for this study was acquired from the ARS Culture Collection (NRRL). Yeast extract-peptone-dextrose (YPD) medium (Sigma-Aldrich, St. Louis, MO) was used for culture of various *L. starkeyi strains.*

### Plasmid construction and gene cloning in *E. coli*

This study utilized the standard protocols for cloning in *E. coli* that were described in our earlier publication [62], with the addition of the newer Gibson Assembly Cloning Kit (NEB, Ipswich, MA, USA) for insertion of target genes into plasmids. Primers used for this study are listed in Table 1. The selected signal peptides (as described below) were fused to the catalytic domains of the cellulase genes, chimeric TeTr*cbh1* and *eg2,* respectively (Fig. 1a), and cloned into expression cassettes for *L. starkeyi* containing native pyruvate kinase (*pyk*) promoter and the galactokinase (*gal1*) terminator (Fig. 1b).

**Table 1:** Primers used in this study

Gene cloning or construction	Primer	Primer nucleotide sequence
Plasmid backbone of pLS1	474_LS1_F1	AGTTTAGAGATGTACAAGGGGTATTGA
	475_LS1_R1	GGCCAGAACGGCCGTGAGAATAGTAAAGGCGGTAGCGAGCTTCATGTTGGCTGTAGTGATACGGACGCAG
*eg2* insertion to pLS1	476_LS1-F2	CTCGCTACCGCCTTTACTATTCTCACGGCCGTTCTGGCCCAACAGACGGTGTGGGGCCAA
	477_LS1-R2	CGCTGACAATGCACCTCAATACCCCTTGTACATCTCTAAACTTTATCACTTGCGGGCAAGGC
	505_EGII_F2	GAGCCCCATGACGTCAACATT
Plasmid backbone of pLS2	474_LS1_F1	AGTTTAGAGATGTACAAGGGGTATTGA
	478_pLS2-R1	GCAATCTCGTCGAAGAATATAACGAGCAACCACAATAGGAGACAGTGATATCACTCCCAGAACAGCGATGAAAAAGTTGATCAGCAACATGTTGGCTGTAGTGATACGGACGCAG
*eg2* insertion to pLS2	479_pLS2_F2	cctattgtggttgctcgttatattcttcgacgagattgcCAACAGACGGTGTGGGGCCAA
	477_LS1-R2	CGCTGACAATGCACCTCAATACCCCTTGTACATCTCTAAACTTTATCACTTGCGGGCAAGGC
Plasmid backbone of pLS3	474_LS1_F1	AGTTTAGAGATGTACAAGGGGTATTGA
	480_LS3_R1	AGCTCCCAAAACCAACGTCGCCAACAGAATGTTAACCAGTACAATCCGTGTGATTGAGGGGACCATTGTAAATATTGAAGGCACGTAGATTAATGTCATGTTGGCTGTAGTGATACGGACGCAG
*eg2* insertion to pLS3	481_LS3_F1	ctggttaacattctgttggcgacgttggttttgggagctCAACAGACGGTGTGGGGCCAA
	477_LS1-R2	CGCTGACAATGCACCTCAATACCCCTTGTACATCTCTAAACTTTATCACTTGCGGGCAAGGC
Plasmid backbone of pLS4	474_LS1_F1	AGTTTAGAGATGTACAAGGGGTATTGA
	475_LS1_R1	GGCCAGAACGGCCGTGAGAATAGTAAAGGCGGTAGCGAGCTTCATGTTGGCTGTAGTGATACGGACGCAG
*cbh1* insertion to pLS4	482_pLS4_F1	GCTCGCTACCGCCTTTACTATTCTCACGGCCGTTCTGGCCCAGCAGGCAGGGACCGCTACC
	483_pLS4_R1	GCTGACAATGCACCTCAATACCCCTTGTACATCTCTAAACTTTATCACAGGCATTGGGAGTAGTAAGGG
Plasmid backbone of pLS5	474_LS1_F1	AGTTTAGAGATGTACAAGGGGTATTGA
	478_pLS2-R1	GCAATCTCGTCGAAGAATATAACGAGCAACCACAATAGGAGACAGTGATATCACTCCCAGAACAGCGATGAAAAAGTTGATCAGCAACATGTTGGCTGTAGTGATACGGACGCAG
*cbh1* insertion to pLS5	484_LS5_F1	cctattgtggttgctcgttatattcttcgacgagattgcCAGCAGGCAGGGACCGCTACC
	483_pLS4_R1	GCTGACAATGCACCTCAATACCCCTTGTACATCTCTAAACTTTATCACAGGCATTGGGAGTAGTAAGGG
Plasmid backbone of pLS6	474_LS1_F1	AGTTTAGAGATGTACAAGGGGTATTGA
	480_LS3_R1	AGCTCCCAAAACCAACGTCGCCAACAGAATGTTAACCAGTACAATCCGTGTGATTGAGGGGACCATTGTAAATATTGAAGGCACGTAGATTAATGTCATGTTGGCTGTAGTGATACGGACGCAG
*cbh1* insertion to pLS6	485_LS6_F1	ctggttaacattctgttggcgacgttggttttgggagctCAGCAGGCAGGGACCGCTACC
	483_pLS4_R1	GCTGACAATGCACCTCAATACCCCTTGTACATCTCTAAACTTTATCACAGGCATTGGGAGTAGTAAGGG
Plasmid backbone of pLS7	474_LS1_F1	AGTTTAGAGATGTACAAGGGGTATTGA
	486_LS7_R1	TGCAGCTCCCAAAACCAACGTCGCCAACAGAATGTTAACCAGTACAATCCGTGTGATTGAGGGGACCATGTTGGCTGTAGTGATACGGACGCAG
*eg2* insertion to pLS7	487_LS7_F1	ggttaacattctgttggcgacgttggttttgggagctgcaCAACAGACGGTGTGGGGCCAA
	477_LS1-R2	CGCTGACAATGCACCTCAATACCCCTTGTACATCTCTAAACTTTATCACTTGCGGGCAAGGC
Plasmid backbone of pLS8	474_LS1_F1	AGTTTAGAGATGTACAAGGGGTATTGA
	486_LS7_R1	TGCAGCTCCCAAAACCAACGTCGCCAACAGAATGTTAACCAGTACAATCCGTGTGATTGAGGGGACCATGTTGGCTGTAGTGATACGGACGCAG
*cbh1* insertion to pLS8	488_LS8_F1	ggttaacattctgttggcgacgttggttttgggagctgcaCAGCAGGCAGGGACCGCTACC
	483_pLS4_R1	GCTGACAATGCACCTCAATACCCCTTGTACATCTCTAAACTTTATCACAGGCATTGGGAGTAGTAAGGG
Correct insertion sequencing	491_ES_F1	GTTGAGCTGCCGCGTGTTCTG
	492_ES_F2	CACACGCGGCTAACACCGATT
	493_ES_R1	CGTTCGATTGGGTTGGTTGGAC
	494_ES_R2	CTGCCACGTCGTTCGATTGGG
eg2 sequencing	497_EGII_F1	CAACAGACGGTGTGGGGCCAA
	498_EGII_R1	TTATCACTTGCGGGCAAGGC
	505_EGII_F2	GAGCCCCATGACGTCAACATT
*cbh1* sequencing	499_CBHI_F1	CAGCAGGCAGGGACCGCTACC
	500_CBHI_R1	TTATCACAGGCATTGGGAGTAGTAAGGG
	506_CBHI_F2	ATACTGGAATCGGCGACCAC

pLS1, 2, 3, 4, 5, 6, 7, and 8 are the plasmids constructed and used in this study (also see Table 3)

### Signal peptide selection

There are two sources for the signal peptides used in this study, which include published articles and our analysis of *L. starkeyi* genome sequence. The signal peptides were identified using SignalP 4.1 (http://www.cbs.dtu.dk/services/SignalP). In this study, a total of four signal peptides were selected and used; these signal peptides can be divided into three types based on their cleavage-site motifs (Table 2): LRR/DCT of *L. starkeyi* amylase, VLG/AAV of *L. starkeyi* dextranase I and II, and VLA/APL protease of *Y. lipolytica.* It was reported that some customized signal peptides used for thermostable cellulase secretion have no observable effect on enzyme performance, whereas others might influence its substrate specificity in *Bacillus subtilis* [40].

**Table 2:** Signal peptides used in this study and their guided recombinant proteins

Signal peptide^a^	Gene accession no.	Sequence of signal peptide^b^	No. of residues	Constructed plasmid and secretion of cellulases^c^	
				EG II	Chimeric *TeTr*CBH I
Protease (spPRO)	P09230	MKLATAFTILTAVLA/APL	15	pLS1 (EG II); Y	pLS 4 (CBH I); Y
Amylase (spAMY)	AY155463	MLLINFFIAVLGVISLSPIVVARYILRR/DCT	28	pLS2 (EG II); Y	pLS 5 (CBH I); Y
Dextranase 1 (spDEX1)	AY520537	MTLIYVPSIFTMVPSITRIVLVNILLATLVLG/AAV	32	pLS3 (EG II); Y	pLS 6 (CBH I); ND
Dextranase 2 (spDEX2)	AAS90631	MVPSITRIVLVNILLATLVLG/AAV	21	pLS7 (EG II); Y	pLS 8 (CBH I); Y

^a^Except the signal peptide of protease (spPRO) that was derived from *Y. lipolytica*, all other signal peptides were derived from *L. starkeyi*

^b^/, cleavage site for a signal peptidase between the signal peptide and the mature protein

^c^For the secretion of cellulases in the cells transformed with the constructed plasmids, Y represents efficient secretion, whereas ND for not detected

**Table 3:** Transformation efficiency of the plasmids studied for *L. starkeyi*

Plasmid	Signal peptide	Cellulase gene	DNA used for transformation (mg DNA)	No. of colonies	No. of colonies/mg DNA
pLS1	spPRO	*eg2*	11.6	66	6
pLS4	spPRO	TeTr*cbh1*	23.6	1024	43
pLS2	spAMY	*eg2*	4.9	44	9
pLS5	spAMY	TeTr*cbh1*	37.5	127	3
pLS3	spDEX1	*eg2*	17.5	154	9
pLS6	spDEX1	TeTr*cbh1*	17.9	48	3
pLS7	spDEX2	*eg2*	23.5	1920	82
pLS8	spDEX2	TeTr*cbh1*	6.5	22	3

*TeTr*CBH I nomenclature: TeTr*cbh1*, chimeric CBH I where the catalytic domain is from *Talaromyces emersonii* CBH I and both linker peptide and CBM1 are from *T. reesei*; *eg2*, *T. reesei* endoglucanase EG II

### Transformation of *L. starkeyi*

The “optimized transformation protocol” described by Calvey et al. [16] was employed in this study. Briefly, cells were grown to an OD_600_ of approximately 10. Cells were then collected via centrifugation, washed with sterile water, and suspended in 500 µL of 0.1 M LiAC. From this cell suspension, 50 µL of cells were transferred to an Eppendorf tube and mixed with transformation buffer and 5 µg of linearized DNA of interest. Transformation mixture consisted of 240 μL 50% PEG (3650), 30 μL 1.0 M LiAc, 15 μL ssDNA, and 15 μL of DNA/H_2_O, to a final volume of 350 μL (including cells) and a final LiAc concentration of 86 mM. The tubes containing the mixture were vortexed to mix and incubated in a 30 °C incubator without shaking for 3 h, and then in a 40 °C water bath for a 5 min heat shock. Following the heat shock, the cells were suspended in 1 mL of YPD and incubated for 4 h at 30 °C with shaking at 220 rpm. The cells were then plated on PYD plates containing 30 μg/mL of clonNAT.

### Protein extraction, SDS-PAGE, and western blot analysis

Cells collected from the selected transformants were grown on YPD broth with 30 μg/mL of clonNAT to reach cell density of OD_600_ 12–15 under the growth condition of 30 °C and 200 rpm. The culture was centrifuged and cells and supernatant were separated. Supernatant supplemented with the protease inhibitor cocktail cOmplete™ (Sigma-Aldrich, Louis, MO, USA) was concentrated and washed into PBS (phosphate-buffered saline, pH 7.4) by centrifugation with 10-kDa cut-off membrane. This concentrated supernatant was used for analysis of SDS-PAGE and western blots, as well as for enzyme activity determination. Protein concentration was measured by Pierce BCA Protein Assay Kit (Thermo Fisher Scientific, Waltham, MA, USA).

For cell-extract protein, cells were broken in liquid nitrogen, and the protease inhibitor cocktail cOmplete™ (Sigma-Aldrich, Louis, MO, USA) was added to the cell extract according to manufacturer’s protocol. The cell extract was washed and concentrated into PBS (pH 7.4) by centrifugation over a 10-kDa cut-off membrane. This concentrated cell extract was used for analysis of SDS-PAGE and western blot. Protein concentration was measured using the Pierce BCA Protein Assay Kit (Thermo Fisher Scientific, Waltham, MA, USA).

### Protein purification

Protein purification of the chimeric CBH I and EG II was conducted using the procedures modified from our recent publication [41], and are described as follows: The harvested culture broths for both enzymes were supplemented with protease inhibitor cocktail of cOmplete (Sigma, Louis, MO, USA) and then concentrated with Tangential Flow Filtration (TFF) using a 10,000 MWCO filter and then buffer-exchanged into 20 mM Bis–Tris buffer pH 6.5 in preparing for chromatographic purification. The concentration of (NH_4_)_2_SO_4_ in the sample was gradually adjusted to 1.5 M, and filtered with a 0.45-µm Nalgene Rapid-Flow Bottle Top filter (Thermo Scientific, Rockford, IL, USA), followed by application to a XK 16/10 hydrophobic interaction chromatography column that packed with the Sepharose 6 fast flow resin in 50 mM Bis–Tris buffer pH 6.5 with a descending 1.5 M (NH_4_)_2_SO_4_ gradient. The initially purified sample was desalted into 20 mM Bis–Tris buffer pH 6.5 by using two HiPrep 26/10 desalting columns (connected in series), and then loaded onto a Tricorn 10/100 anion exchange column packed with Source 15Q resin in 20 mM Bis–Tris buffer, pH 6.5, and an increasing NaCl gradient (0–300 mM). The final, purified sample was acquired using the gel filtration on a 26/60 Superdex 75 column in 20 mM acetate buffer pH 5.0 with 100 mM NaCl. When needed, the samples were concentrated by using Vivaspin 20 10-kDa concentrators, and the desired protein fractions were identified by using a para-nitrophenyl-β-d-lactopyranoside assay [63, 64] for CBH I or the K-CELLG3 endo-cellulase assay (Megazyme, Bray, Ireland) for EG II. Concentration of the purified recombinant proteins was measured by Nanodrop 100 spectrophotometer (Thermo Scientific, Wilmington, DE, USA).

### Genomic DNA extraction and estimation of transgene copy number

Genomic DNA of the representative transformants and the wild-type control strain was isolated from *L. starkeyi* cell pellets using the ZR Fungal/Bacterial DNA Miniprep kit (cat.# D6005; Zymo Research, Irvine, CA), and the bead beating was conducted using the Qiagen Tissuelyser II with a frequency set at 30/s for 5 min. The concentration of extracted genomic DNA was measured using Nanodrop, and adjusted to 20 ng/µL and stored at −80 °C.

Real-time quantitative PCR (qPCR) has been widely used to determine gene copy number in engineered organisms [65, 66]. In this study, the copy number of expressed chimeric *cbh1* and *eg2* genes in the transformants were estimated according to a method described by Weng et al. [59], which has been used in numerous studies [66–68]. For comparison, the endogenous eukaryotic initiation factor 5 (eif5) gene was used as a single-copy reference gene [69]. The primers for these genes were listed in Additional file 2: Table S2.

Relative standard curves for target and reference genes were established with 4-log-range dilutions [70, 71] of each transformant’s genomic DNA, i.e. 2, 0.2, 0.02, and 0.002 ng/µL, which led to 10, 1, 0.1, and 0.01 ng per PCR reaction. The threshold cycle or Ct values were plotted against the log-transformed genomic DNA concentration of each dilution, and the amplification efficiencies (E) were calculated based on slopes of relative standard curves, using the formula E = 10^(−1/S)^ − 1. The copy-number (X_0_/R_0_) of target gene (X) versus reference gene (R) was calculated using the equation 5 described by Weng et al. [59]. For the examined transformants, triplicates of extracted genomic DNA were performed and the copy numbers were presented as the mean ± standard error of the mean (SEM).

Real-time qPCR was performed in 96-well plates using an ABI 7300 Real-Time PCR System (Thermo Fisher Scientific, Applied Biosystems, Grand Island, NY). qPCR reactions were performed in a final volume of 20 µL, containing 10 µL of 2 × Power SYBR Green PCR Master Mix (Cat. no. 4367659, Thermo Fisher Scientific, Applied Biosystems, Grand Island, NY), 2.5 µL of 4 µM each of forward and reverse primers, and 5 µL of template DNA. The qPCR cycling were set at 50 °C for 2 min, 95 °C for 10 min followed by 36 cycles of 95 °C for 15 s, 60 °C for 1 min. A final disassociation step was 95 °C for 15 s, 60 °C for 1 min, 95 °C for 15 s, and 60 °C for 15 s. PCR reactions were performed in triplicate.

### Assay for the enzyme activity of crude enzyme (secretome)

Activities of the crude enzyme preparations were assayed as the ability to saccharify the cellulose fraction of a model industrial lignocellulosic substrate, dilute-acid-pretreated corn stover (PCS). The standard biomass substrate used in the activity assays was NREL dilute-acid-pretreated corn-stover P050921, produced in a vertical pulp digester supplied by Sunds Defibrator (now Metso Corporation, Helsinki) as described earlier [72], with a residence time of approximately 1 min at 190 °C with 0.45 g H_2_SO_4_ per g dry biomass at 30% solids loading, to yield a material 59.1% in glucan, 5.1% in xylan and 25.3% in lignin. This biomass material was loaded into the digestion mixtures at 5 mg glucan per mL. The tested crude enzyme preparation was then loaded at a ratio of 120 mg total protein per g biomass glucan, as part of a cocktail also containing the catalytic domain of *A. cellulolyticus* endoglucanase (E1, Y245G mutant (68)) at 1.89 mg/g biomass cellulose and purified *Aspergillus niger* β-glucosidase at 0.5 mg/g biomass cellulose, to form a mixture containing the essential elements of a “full” cellulase system. Assays were conducted at 40 °C and pH 5.0 (20 mM acetate with 0.02% sodium azide), in sealed HPLC vials with continuous mixing by inversion while in a water bath. Representative samples of solid and liquid were withdrawn at different times during the digestion, diluted with water into sealed HPLC vials, boiled 12 min to inactivate enzymes and terminate the reactions, then filtered and analyzed by HPLC to measure released glucose and cellobiose. The total sugar-release numbers were used, along with the known quantity of substrate cellobiose added, to calculate percent conversion of cellobiose.

### Assay for the enzyme activity of purified CBH I protein

Deacetylated and subsequently dilute-acid-pretreated corn stover (DACS) was used as substrate to measure the enzymatic conversion of glucan [73]. The purified CBH I was loaded at 28 mg enzyme per g glucan of DACS supplemented with *A. cellulolyticus* E1-CAT (Y245G) (1.89 mg/g glucan), and *A. niger* β-d-glucosidase (0.50 mg/g glucan).

Assays were carried out in triplicate in a buffer containing 20 mM acetate buffer pH 5.0 with 0.02% (w/v) sodium azide. The initial digestion volume of 1.7 mL was put into crimp-sealed 2.0-mL HPLC vials, with constant mixing by inversion at 10 rpm in a 40 °C water bath. Representative 0.1-mL aliquots of liquid and solids were withdrawn from the vials for analysis at designated time points during the digestions. The obtained aliquots of digestion mixture were diluted 18-fold (with deionized water) into sealed 2.0-mL HPLC vials, followed by immersion for 10 min in a boiling water bath to terminate the enzymatic reaction. The diluted digestion-mixture aliquots were filtered with 0.2-μm filter before the determination of released sugars by HPLC, as described previously [41].

### Assay for the enzyme activity of purified EG II protein

Endocellulase activity of the recombinant EG II was measured at 40 °C and pH 4.5 (acetate) with the Cellulase Assay Kit (CELLG3 Method) (Megazyme, Bray, Ireland) following the procedure provided by the manufacturer.

### Deglycosylation analysis of purified CBH I proteins

Endoglycosidase H (Endo H, cat# P0702L; New England Biolabs, Ipswich, MA) was used to remove N-linked carbohydrates from purified TeTrCBH I generated in this study, as well as the control CBHI, which was the native TrCBH I purified from *T. reesei* Rut-C30. TeTrCBH I and TrCBH I proteins were treated with Endo H for 20 h at 37 °C according to the manufacturer’s instructions. In the parallel treatment without Endo H (that is, -Endo H), Endo H was replaced by water. Protein samples were separated by SDS-PAGE using Invitrogen NuPAGE Novex 12% Bis–Tris Mini Gel and visualized with colloidal Coomassie blue staining.

## Additional files

**Additional file 1: Table S1.** The annotation of the putative proteins and the identification of its CAZY family members in the genome of *Lipomyces starkeyi*. **Sheet 1**, the annotation of the putative proteins in the genome of *L. starkeyi;* **Sheet 2**, the identification of its CAZY family member proteins.

**Additional file 2: Table S2.** Primer sequences for real-time qPCR. **Table S3.** Relative standard curve for target (chimeric CBH I and EG II) and reference (EIF5) genes. **Figure S1.** Chimeric CBHI activities of crude protein mixtures (secretomes) produced by strains having different copy numbers and using different signal peptides to guide secretion of TeTrCBH I.

## Abbreviations

CBP: consolidated bioprocessing
E: amplification efficiency
EG II: endoglucanase II
*gal1*: galactokinase gene
GH: glycoside hydrolase
PBS: phosphate-buffered saline
*pyk*: pyruvate kinase gene
SSF: simultaneous saccharification and fermentation
spAMY: signal peptide for amylase
spDEX1: signal peptide for dextranase 1
spDEX2: signal peptide for dextranase 2
spPRO: signal peptide for protease
tdh3: glyceraldehyde-3-phosphate dehydrogenase gene
TeTrCBH I: chimeric cellobiohydrolase I formed by fusion of the catalytic domain from *Talaromyces emersonii* CBH I with the linker peptide and cellulose-binding domain from *T. reesei* CBH I
YPD: yeast extract-peptone-dextrose
