# Supplementary material for: Expression and secretion of fungal endoglucanase II and chimeric cellobiohydrolase I in the oleaginous yeast Lipomyces starkeyi
Source: Microb Cell Fact. 2017 Jul 24;16:126. doi: 10.1186/s12934-017-0742-5 (PMC5525229; doi:10.1186/s12934-017-0742-5)
Supplement: Supplementary file 2 — Additional file 2: Table S2. Primer sequences for real-time qPCR. Table S3. Relative standard curve for target (chimeric CBH I and EG II) and reference (EIF5) genes. Figure S1. Chimeric CBHI activities of crude protein mixtures (secretomes) produced by strains having different copy numbers and using different signal peptides to guide secretion of TeTrCBH I. [file 12934_2017_742_MOESM2_ESM.pdf]

Title: Expression and secretion of fungal endoglucanase II and chimeric cellobiohydrolase I in the oleaginous yeast *Lipomyces starkeyi*

Additional file 2

File type: PDF file (.pdf)

Supplemental Table S2. Primer sequences for real-time qPCR.

Supplemental Table S3. Relative standard curve for target (chimeric CBH I and EG II) and reference (EIF5) genes.

Supplemental Figure S1. Chimeric CBHI activities of crude protein mixtures (secretomes) produced by strains having different copy numbers and using different signal peptides to guide secretion of TeTrCBH I.

Supplemental Table S2. Primer sequences for real-time qPCR.

Target genes	Primer sequences	Amplicon size (bp)
CBH I	CBH I-2-F: TTCACCGCATCTAACCCACC	159
	CBH I-2-R: GGTAGTTCCAGACGCACACA	
EG II	EG II-3-F: TGACCATCTTCCGACTCCCT	136
	EG II-3-R: ACGATGCAGTAAGCACCCAA	
EIF5	EIF5F2: ACACTAGCCGGAGGGTTTTT	237
	EIF5R2: AGCTACGGACCGATACATGG	

Supplemental Table S3. Relative standard curve for target (chimeric CBH I and EG II) and reference (EIF5) genes.

**Relative standard curves**	**Amplification efficiency (E)**	**Slope (S)**	**y-intercept (I)**	**R^2**
EIF5 using LS2-1 genomic DNA template	0.99	-3.34	19.363	0.9997
EG II using LS2-1 genomic DNA template	0.91	-3.57	16.970	0.9974
EIF5 using Ls4-8 genomic DNA template	0.89	-3.610	17.733	1.0000
CBH II using Ls4-8 genomic DNA template	0.92	-3.52	15.802	0.9998
EIF5 using Ls8-7 genomic DNA template	0.90	-3.599	17.816	0.9998
CBH II using Ls8-7 genomic DNA template	0.94	-3.47	15.123	0.9991
EIF5 using Ls5-10 genomic DNA template	0.93	-3.51	17.740	0.9998
CBH II using Ls5-10 genomic DNA template	0.99	-3.35	15.845	0.9997

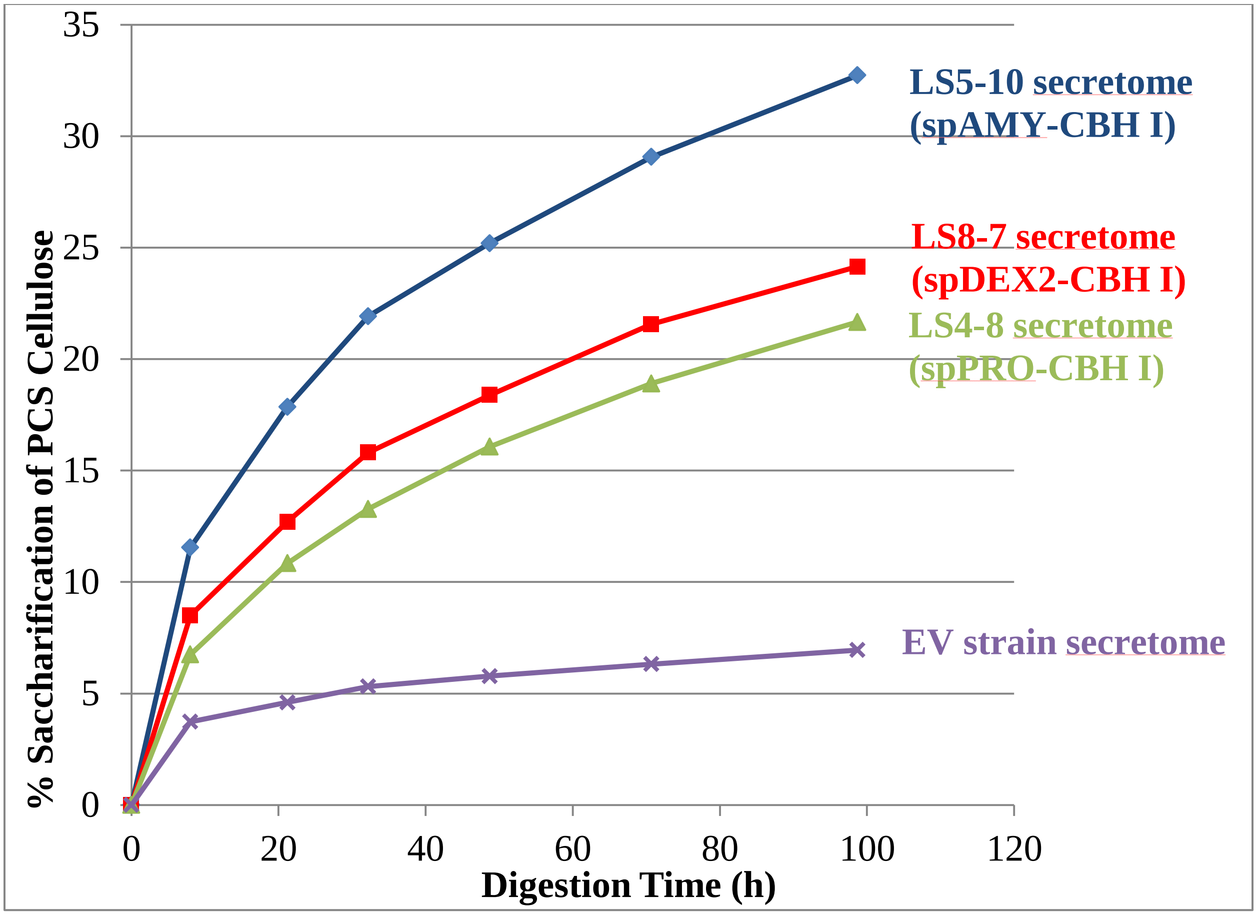

Supplemental Figure S1. Chimeric CBHI activities of crude protein mixtures (secretomes) produced by strains having different copy numbers and using different signal peptides to guide secretion of TeTrCBH I. Strain LS5-10 contains 2 copies coding for the spAMY signal peptide from *L. starkeyi amylase* (Gene-accession no. AY155463, Table 2) to express the chimeric CBH I; LS8-7 and LS4-8 use single copies of the signal peptides from *L. starkeyi* dextranase 2 (spDEX2) and Y. lipolytica protease (spPRO) respectively. Progress curves illustrate the saccharification of the cellulose content of dilute-acid-pretreated corn stover (loaded at 5 mg/mL) at 40°C and pH 5.0 in 20 mM acetate. In all digestions, total secreted protein was loaded at 120 mg/biomass cellulose. In addition to the (washed) secretome proteins, each digestion mixture contained as "helper" enzymes the catalytic domain of *A. cellulolyticus* endoglucanase (E1, Y245G mutant (68)) at 1.89 mg/ g biomass cellulose and purified *A. niger* β-glucosidase at 0.5 mg/g biomass cellulose. EV, empty vector.
